# L-Ascorbic Acid Shapes Bovine *Pasteurella multocida* Serogroup A Infection

**DOI:** 10.3389/fvets.2021.687922

**Published:** 2021-07-08

**Authors:** Guangfu Zhao, Pan Li, Hao Mu, Nengzhang Li, Yuanyi Peng

**Affiliations:** ^1^Chongqing Key Laboratory of Forage and Herbivorce, College of Veterinary Medicine, Southwest University, Chongqing, China; ^2^Key Laboratory for Bio-Resource and Eco-Environment of Education of Ministry, The Center for Growth, Metabolism and Aging, College of Life Sciences, Sichuan University, Chengdu, China; ^3^Chongqing Academy of Animal Science, Chongqing, China

**Keywords:** Bovine *Pasteurella multocida* serogroup A, pneumonia, metabolomics, macrophage, L-ascorbic acid

## Abstract

Bovine *Pasteurella multocida* serogroup A (bovine PmA) is one of the most important pathogens causing fatal pneumonia in cattle. However, it is largely unknown how nutrition shapes bovine PmA infection. Here, we discovered that the infected lung held the highest bacterial density than other tissues during infection. By screening the different metabolites between high (lung)- and low (liver)-bacterial density tissues, the present work revealed that L-ascorbic acid and L-aspartic acid directly influenced bovine *P. multocida* growth. Interestingly, L-ascorbic acid, which is expressed at higher levels in the infected livers, inhibited bovine PmA growth as well as virulence factor expression and promoted macrophage bactericidal activity *in vitro*. In addition, ascorbic acid synthesis was repressed upon bovine PmA infection, and supplementation with exogenous L-ascorbic acid significantly reduced the bacterial burden of the infected lungs and mouse mortality. Collectively, our study has profiled the metabolite difference of the murine lung and liver during bovine PmA infection. The screened L-ascorbic acid showed repression of bovine PmA growth and virulence expression *in vitro* and supplementation could significantly increase the survival rate of mice and reduce the bacterial load *in vivo*, which implied that L-ascorbic acid could serve as a potential protective agent for bovine PmA infection in clinic.

## Introduction

*Pasteurella multocida* (Pm) is a pathogenic Gram-negative bacterium with multiple host types, like chicken, pig, rabbit, and cattle. Clinically, Pm infection was mainly characterized by fowl cholera, swine atrophic rhinitis, rabbit septicemia, bovine pneumonia, etc. (1). Sometimes, humans can even be infected with Pm *via* animal bites and scratches (2). According to the capsular characteristics, Pm can be classed into A, B, D, E, and F serogroups (3). Among them, bovine *P. multocida* serogroup A (bovine PmA) is one of the most important pathogenic bacteria causing bovine pneumonia, with high morbidity and mortality around the world, which has led to immense economic losses in animal husbandry (4–6).

Generally, the most severely injured tissue is often accompanied by higher bacterial density (7–9). Cattle infected with bovine PmA usually exhibits serious pneumonia in clinic, and it was found that profuse growth of bovine PmA existed in the bovine lungs (10). A potential cause to this situation appears to be that the bactericidal activity of alveolar macrophage is weaker than peritoneal macrophage when exposed to PmA (11). Colonization is the prerequisite for infection, and various conditions can affect bacterial colonization, like host immunity, nutrition, bacterial virulence factors, and so on. For example, bacterial adhesin, an important bacteria virulence factor, is one prerequisite for bacterial colonization, which owns a precise selectivity for target molecules and recognizes molecular motifs in a lock and key mode (12). Another is that enterotoxigenic *Escherichia coli* (ETEC) strains need K88 fimbrial adhesin to adhere to the front of the piglet small intestine and then release enterotoxins to cause piglet diarrhea (13, 14). Recently, increasing researches demonstrated that nutrition plays a key role in bacterial infection. Haber and his co-workers reported that *Listeria monocytogenes* loads in tissues as well as the expression of virulence genes are dependent on L-glutamine levels (15). Likewise, *Campylobacter jejuni* loads are influenced by microbiota-derived short-chain fatty acid (16).

In our former works, we have found that some nutrition, particularly amino acids, played important roles in bovine PmA infection based on the mouse model (17–20). Considering that pneumonia is the most typical symptom of bovine PmA infection, the present work has found that the infected lungs had the highest bacterial density when compared with other organs. Then, metabolomics was employed to compare the metabolite profile difference between the high- and low-bacterial density tissues. Finally, this study has demonstrated that L-ascorbic acid (AA) shaped bovine PmA infection and AA could serve as a protective agent for anti-bovine PmA infection.

## Materials and Methods

### Bacterial Strains and Cultivation Condition

The bovine *P. multocida* serogroup A strains CQ2 (GenBank accession number: No. CP033599) and CQ6 (GenBank accession number: CP033600) were isolated in Chongqing and cultured on Martin's broth agar with 5% horse serum at 37°C (21). *Escherichia coli* DH5α was bought from a commercial company (Tiangen, Beijing, China) and stored in our laboratory. Bovine mastitis *E. coli* and *Salmonella typhimurium* were isolated from ill cattle in Chongqing. Mouse peritoneal macrophages were cultivated in Dulbecco's modified Eagle medium (DMEM) high glucose (hyclone) supplemented with 10% fetal bovine serum (GIBCO) at 37°C under 5% CO_2_.

### Animal Experiment

All animal experiments in this study have been granted permission by the Ethics Committee of Southwest University and likewise adopted the principles of Laboratory Animal Care of the National Institutes of Health, China (Permit No.11-1025). Female Kunming mice (6–8 weeks old, weighing 18–22 g) were purchased from the Institute of Chongqing Herb Medicine. Mice were kept in independently airy cages, lighting cycle at 12 h/day, keeping relative humidity at 50–60% as well as temperature at 20–30°C. For all infection experiments in this study, mice were infected with 10^4^ CFU log-phase growth PmCQ2 *via* intramuscular, intraperitoneal, or intranasal infection. Notably, 1.5% pentobarbital sodium was used to anesthetize mice before intranasal infection. After finishing infection experiment, mice were euthanized to collect their tissues.

### Bacterial Colonization

Collected tissues were homogenized aseptically in 1 ml of saline and diluted to the appropriate gradient in saline to plate in triplicate on Martin's broth agar. Bacterial colonization was recorded *via* counting the average of CFU on this agar after 20-h cultivation at 37°C.

### Histopathological Examination

For histopathological examination, tissues were promptly soaked in 4% paraformaldehyde for 36 h. These tissues then were dehydrated using gradient ethanol and embedded in paraffin. Afterwards, these paraffins were cut at 3 μm thick and stained with hematoxylin and eosin (H&E). H&E staining was estimated by three different researchers.

### Sieving Metabolites

BacTrac™ 4300 Microbiological Analyzer (Sylab, Austria) was applied to analyze the influence of each metabolite on the bacterial growth. This apparatus can reflect the change of the bacterial growth *via* measuring impedance (22). Briefly, an approximate dose of 10^4^ CFU bacterial grown to log-phase was added rapidly into an aseptic test vial containing 8 ml of Martin's broth only or supplemented with a certain metabolite. Each group consisted of five individual test vials with the same treatment. Then, the vials were incubated at 37°C for more than 12 h, and a real-time impedance curve was given. The impact of one metabolite on bacterial growth can be reflected by the real-time impedance curve. The change of pH after adding a certain metabolite was adjusted by sodium hydroxide or hydrochloric acid.

### Measurement of Bacterial Growth Curve

Bacterial growth curves were measured *via* the optical density value (OD_600_) or the plate-counting method. In brief, an approximate dose of 10^9^ CFU bacteria grown to log-phase was rapidly added to an aseptic flask containing 100 ml of Martin's broth only or supplemented with a certain metabolite (*n* = 3 per group). The flasks were put into a shaker set to 37°C and speed at 220 rpm. Every 2 or 3 h, 1 ml of Martin's broth was taken aseptically and diluted to an appropriate dilution to estimate the optical density at 600 nm *via* using Vis Spectrophotometer (JINGHUA, China) or measure the bacteria number *via* the plate-counting method. Growth curves were drawn dependent on the results of different time points. The pH values were adjusted as above.

### Q-PCR

Bacteria, cell, and tissue RNA were extracted using the total cell or tissue RNA extraction kit from Tiangen (China) and stored at −80°C, and RNA sample reverse transcription into cDNA used a FastKing reverse transcription kit from Tiangen (China). All operations were carried out according to the manufacturer's instruction. Q-PCR reaction was carried out with 1 μg of cDNA, 200 nM primer, and 5 μl of Bio-rad SYBR enzyme (USA) in a final volume of 10 μl by Bio-rad CFX96 machine. All primer sequences are available in Supplementary Table 1.

### Isolation of Mouse Peritoneal Macrophage

Isolation of mouse peritoneal macrophage was manipulated as reported by Dey with some modifications (23). Briefly, mice received once intraperitoneal injections with 1.5 ml of 4% thioglycolate broth to stimulate macropahges biogenesis for 4 days. Afterwards, mice were euthanized and 4 ml of fresh DMEM high glucose medium was intraperitoneally injected to collect peritoneal lavage fluid. Centrifugation of the obtained peritoneal lavage solution at 1,800 rpm for 3 min and supernatant was removed. Finally, cells were resuspended with DMEM high glucose medium containing 10% fetal serum and these cells were ready to be used. Additionally, flow cytometer examination indicated that more than 90% cells isolated were positive for CD11b, a monocyte or macrophage marker, indicating that these cells belong to peritoneal macrophages.

### Cell Viability Test

The cell viability test was performed by CCK-8 kit (Solarbio) according to the manufacturer's instruction. Briefly, suspension cells were added to a 96-well plate to ~2,000 cells per well, and cultivated for 2 h at 37°C under 5% CO_2_ to allow cell adhesion, and washed three times with phosphate buffered saline (PBS, hyclone). Then, 10 μl of analyzed substance or saline was added to the experimental group, black group, and negative control group, respectively, and incubated for another 6 h. After that, the cells were washed three times with PBS, adding 10 μl of CCK-8 solution per well, and cells were incubated for another 4 h. Finally, the optical density at 450 nm was measured using a Microplate Reader (Thermo). Each group contains five repeats and the black group has no cell. The cell viability was estimated *via* the following formula:


$$\begin{array}{l} \text{Cell viability }(\%)\;=\;(\text{experimental group}\\ \;-\text{black group})\text{/}(\text{negative control }-\text{ black group})*100. \end{array}$$

### Phagocytosis and Intacellular Survival Assays

Phagocytosis assay was performed as described with some modifications (24). Mouse peritoneal macrophage of ~2 × 10^5^ cells were seeded per well in a 24-well plate. Bacteria grown to log-phase were harvested, washed, and resuspended in DMEM high glucose, and added to cells at a multiplicity of infection (MOI) of 1, as well as AA and Asp. After 2-h incubation, cells were rinsed three times with PBS and then added to DMEM high glucose containing 100 μg/ml ciprofloxacin solution for another 0.5 h to kill extracellular bacteria. Then, after rinsing three times with PBS, cells were lysed with 500 μl of PBS containing 0.1% Triton X-100 to release intracellular bacteria. The phagocytized bacteria were enumerated by the plate-counting method.

Intracellular survival analysis was carried out based on a previous report with some modifications (25). The procedures were the same as the phagocytosis assay except that AA, Asp, or saline was added to the medium after killing extracellular bacteria. The surviving bacteria were recorded by the plate-counting method. All assays have three independent experiments.

### Biofilm Formation Assay

Biofilm assay was based on Anchanee's report with some modifications (26). Bacteria grown to log-phase in 100 μl of fresh Martin's broth supplemented with/without a substance were added to a 96-well plate to about 10^7^ CFU per well. The plate was incubated at 37°C for 48 h, and then it was washed three times with PBS. Next, each well was fixated with 100 μl of methanol for 15 min and methanol was removed. One hundred milliliters of 1% Crystal violet solution was added per well to dye cells for 5 min. After that, wells were washed with flowing water and dried at 37°C. Lastly, 100 μl of 33% glacial acetic acid solution was added to each well and their optical density was recorded using a Microplate Reader (Thermo) at OD_590_.

### AA Concentration Assay

Tissue AA concentration assay was operated using an AA assay kit (Sinobestbio, China) according to the manufacturer's instruction. In brief, tissues were mixed with solution buffer at 0.1 g/ml, homogenized on ice, and centrifugated for 20 min at 8,000 *g* under 4°C. Supernatant was collected and the optical density was measured using a Microplate Reader (Thermo) at OD_534_. According to the standard curve, the tissue AA content can be figured out.

### Statistical Analysis

GraphPad Prism software (Prism 6.0) and PASW Statistical 18.0 software (SPSS) were applied for statistical analyses. A group of individual data was expressed as means ± standard error of the mean (SEM), while the remainder of the data were expressed as means ± standard deviation (SD). Mice survival curves were assessed based on Kaplan–Meier analysis (Prism 6.0). Unpaired Student's *t*-test (Prism 6.0) was used to study the data between the two groups, while one-way ANOVA (SPSS) was applied to examine the data among more than two groups. The determinant of whether there is a significant difference is dependent on *P* < 0.05, and *, **, and *** represent *P* < 0.05, *P* < 0.01, and *P* < 0.001, respectively.

## Results

### Bovine *P. multocida* Serogroup a Infection Causes Fatal Pneumonia in a Mouse Model

**Figure 1:**
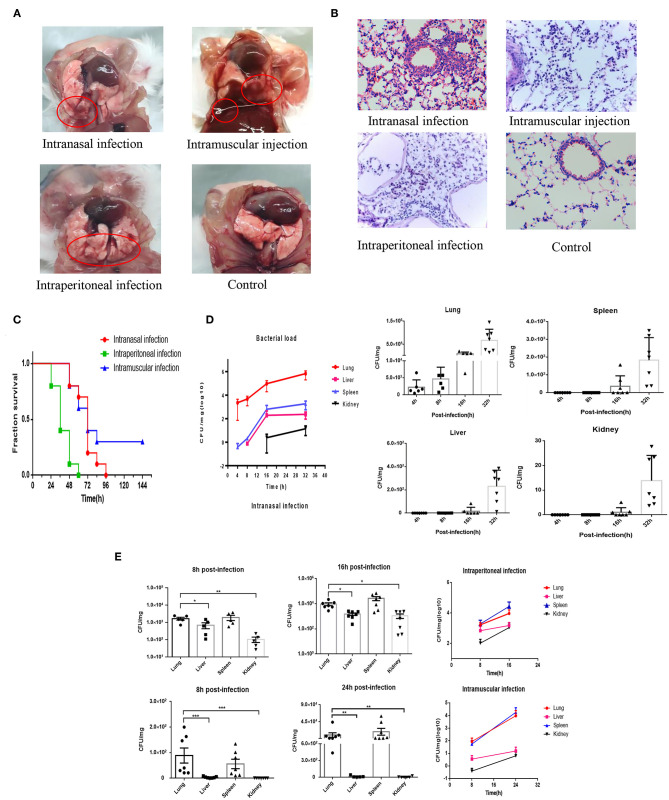
PmCQ2 infection causes fatal pneumonia in mouse models and the infected lung has higher bacteria density than other tissue. **(A,B)** The lung pictures and H&E stainings (×400) of mice that were intranasally infected, intramuscularly infected, or intraperitionally infected with 10^4^ CFU log-phase PmCQ2 for 20 h, and the mice intranasally injected with saline (Control). The red circle indicates the lung damage and pulmonary effusion. **(C)** Survival rates of mice intranasally infected, intramuscularly infected, and intraperitionally infected with 10^4^ CFU log-phase PmCQ2. **(D)** A time-course assay reflecting the lung, liver, spleen, and kidney bacterial density of mice intranasally infected with 10^4^ CFU log-phase PmCQ2. Every single point represents one individual. **(E)** A time-course assay reflecting the lung, liver, spleen, and kidney bacterial density of mice intraperitoneal or intramuscular infected with 10^4^ CFU log-phase PmCQ2. Every single point represents one individual. *, **, and *** represent *P* < 0.05, *P* < 0.01, and *P* < 0.001, respectively.

Clinically, the phenotype of bovine PmA infection is fatal pneumonia in cattle. Nevertheless, considering the price of using cattle as experimental model is too high and few SPF cattle are available today; our lab has successfully established a mouse model to mimic bovine PmA that caused pneumonia (20, 27). In this study, mice infected with bovine PmA (PmCQ2) always present some typical respiratory symptoms, such as cough and abdominal respiration. Likewise, H&E staining also shows serious lung damage and immune cell infiltration, such as lymphocyte and neutrophil, which indicates that bovine PmA has indeed caused a severe inflammatory reaction in the lung (Figures 1A,B). Additionally, mice infected with PmCQ2 exhibited high mortality rate, especially through intraperitoneal injection (Figure 1C). Together, these data suggested that mice infected with bovine PmA indeed induced fatal pneumonia.

### The Lung Has the Highest Bacterial Density Compared to Other Organs Under Bovine PmA Infection

Next, considering the pneumonia phenotype caused by bovine PmA, we suggested that the lung might hold higher bacterial density than other tissues. As shown in Figures 1D,E, the infected lung has the highest bacterial density under intranasal infection, but the lung and the spleen equally contain the highest bacterial density under intraperitoneal infection and intramuscular infection. Notably, the heart blood PCR suggested that bovine PmA infection causes bacteremia (Supplementary Figure 1). Given the spleen has a blood filter function, it is unknown whether the bovine PmA was detained in the spleen or was subjected to colonization. Thus, this study regards the lung as the highest bacterial density tissue (28). Additionally, low bacteria density, if any, was detected in the lung intraperitoneally infected with *E. coli* DH5α, bovine mastitis *E. coli*, and *S. typhimurium* (Supplementary Figure 2), indicating that lung colonization is a feature of bovine PmA. Together, these data suggested that bovine PmA infection resulted in the lung having the highest bacterial density compared to other tissues.

### It Is Found That AA and Asp Influence the Bovine PmA Growth *via* Comparing High- and Low-Bacterial Density Tissues Using Metabolomics

**Figure 2:**
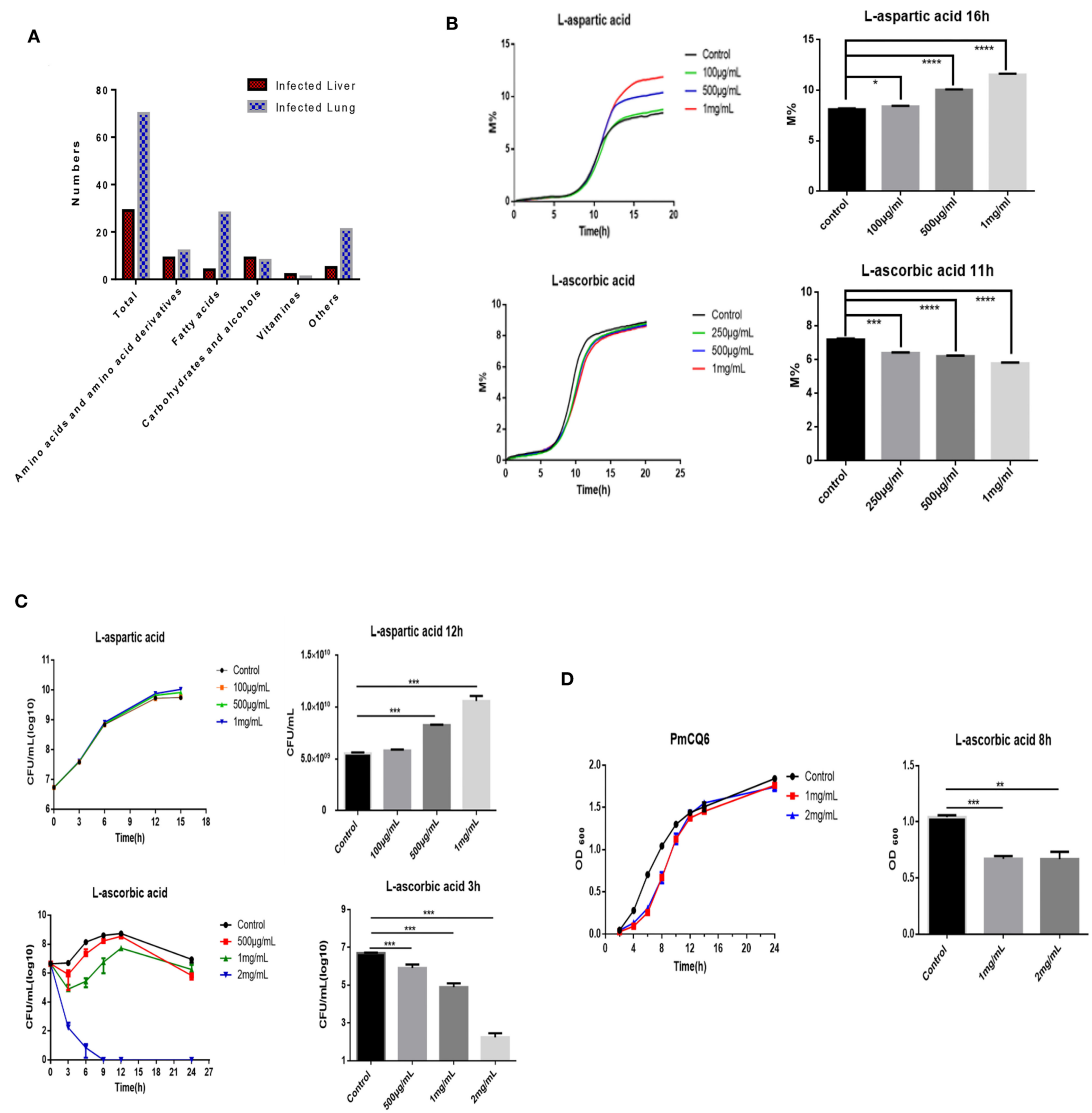
AA and Asp influenced bovine *P. multocida* growth in *vitro*. **(A)** The differently expressed metabolites of the mouse infected lung and liver. These mice were intranasally infected with 10^4^ log-phase PmCQ2 for 16 h and a total of eight mice were analyzed. **(B)** PmCQ2 impedance curves under different doses of AA or Asp supplementation. **(C)** PmCQ2 growth curves under different doses of AA or Asp supplementation. **(D)** PmCQ6 growth curves under different doses of AA. All experiments own three repeats for each group. *, **, ***, and **** represent *P* < 0.05, *P* < 0.01, *P* < 0.001, and *P* < 0.0001, respectively.

Many factors can influence bacterial density, including bacterial virulence factor, host immunity, nutrition, and so on (11, 13–15). To find some potential nutrition that shapes bovine PmA infection, we compared the metabolite profiles of the infected lung and liver using metabolomics. As shown in Figure 2A and Supplementary Figure 3, the metabolomics model is stable and credible and presents a sum of 99 differentially expressed metabolites, 70 in the infected lung and 29 in the infected liver. Next, a number of differentially expressed metabolites were screened whether they directly influence PmCQ2 growth *in vitro*. Although most of the differentially expressed metabolites had no effect on PmCQ2 growth, fortunately, it was found that AA and Asp inhibited and promoted PmCQ2 growth in a dose-dependent manner, respectively (Figures 2B,C and Supplementary Figure 4). Moreover, metabolomics data show that AA was expressed more in the infected liver while the Asp is expressed more in the infected lung, which is consistent with their effects on PmCQ2 (Supplementary Table 2). In addition, AA also inhibited PmCQ6 growth, indicating that the inhibitory effect is conservative among bovine PmA (Figure 2D). Together, these data indicated that both AA and Asp have direct functions on bovine PmA growth.

### AA Inhibits PmCQ2 Virulence Factor Expression and Bovine PmA Infection Leads to AA Deficiency

**Figure 3:**
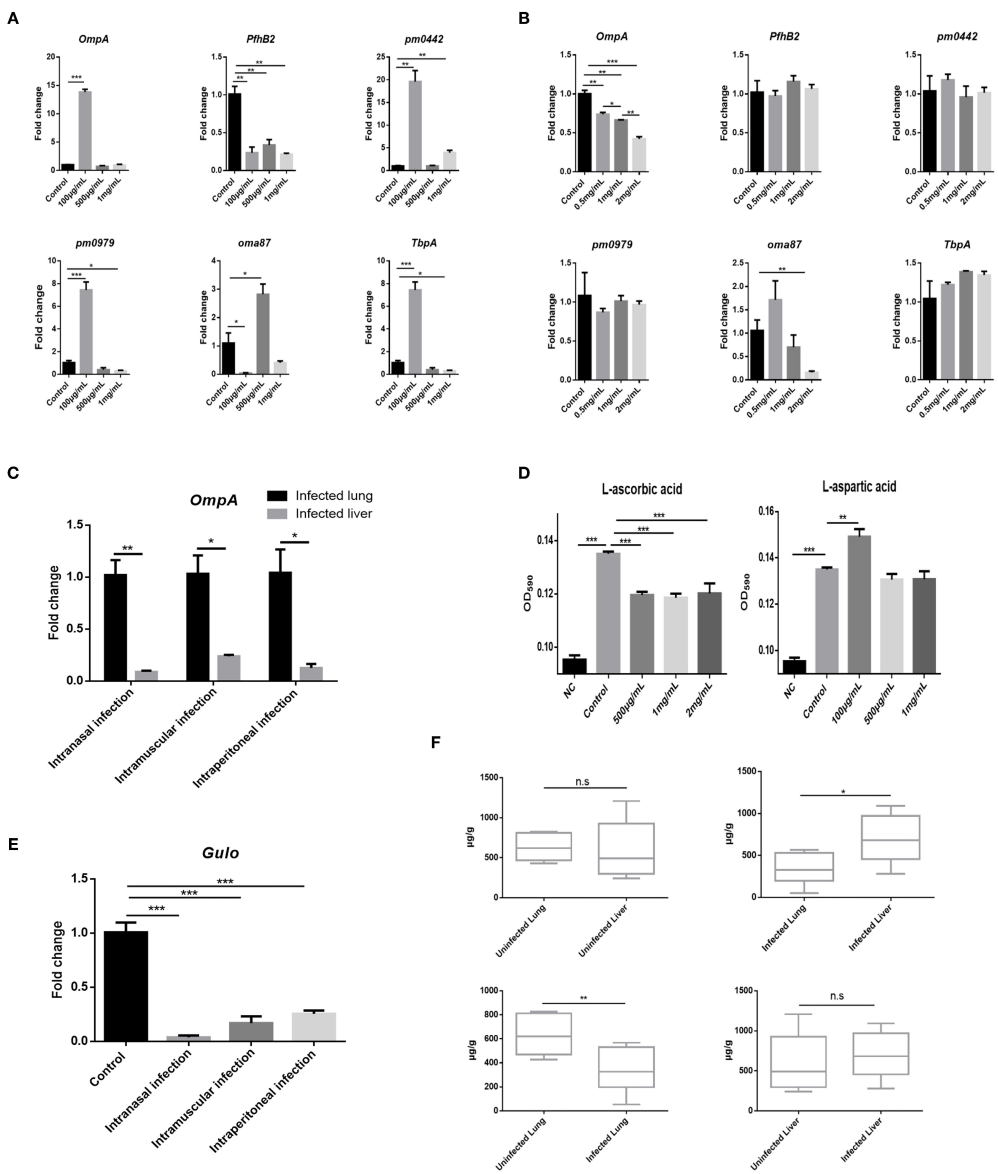
AA inhibited PmCQ2 virulence factor expression and its concentration was downregulated in the lung after PmCQ2 infection. **(A)** Analysis of some PmCQ2 virulence gene expression under different doses of L-aspartic acid. **(B)** Analysis of some PmCQ2 virulence gene expression under different doses of AA. Three repeats for each group. **(C)** The virulence gene, *OmpA*, expressions in the mice lung before infection or after 10^4^ CFU log-phase growth PmCQ2 infection for 16 h. Three repeats for each group. **(D)** PmCQ2 biofilm formation treated with different doses of AA or L-aspartic acid. Five repeats for each group. **(E)** The AA biogenesis gene *Gulo* expressions in the mice lung before infection or after receiving 10^4^ CFU log-phase growth PmCQ2 infection for 16 h. Three repeats for each group. **(F)** Comparison of AA concentrations in health mice lung and liver with mice intranasally infected with 10^4^ CFU log-phase growth PmCQ2 for 32 h. Six repeats for each group. *, **, and *** represent *P* < 0.05, *P* < 0.01, and *P* < 0.001, respectively.

Given bacterial virulence expression is important for colonization, whether AA and Asp could influence bovine PmA virulence gene expression was investigated. As shown in Figures 3A,B, Asp showed no impact on the virulence gene expression, whereas AA downregulated two virulence genes, *OmpA* and *oma87*, in a dose-dependent manner. Interestingly, in agreement with the metabolomics data, less AA was found in the infected lung than the liver with more *OmpA* expression (Figure 3C). In addition, it is found that AA significantly decreased PmCQ2 biofilm biogenesis while Asp promoted PmCQ2 biofilm biogenesis at low dosage (Figure 3D). It is well-known that biofilm is an important virulence factor for bacterial invasion to avoid host immunity system and drug (29). The above data implied that AA may restrain bovine PmA infection *via* inhibiting bacterial virulence expression. To identify whether bovine PmA would disrupt AA biogenesis, the expression of *Gulo*, a gene responsible for AA biogenesis in mice and cattle, was measured. As shown in Figure 3E, *Gulo* expression was inhibited by bovine PmA infection, indicating that AA biogenesis was damaged. In agreement with the *Gulo* expression result, AA deficiency was further evidenced by the downregulation of the AA concentrations in the infected lung (Figure 3F). Moreover, the level of AA in the infected lungs was significantly lower compared with the livers, which was consistent with the metabolomics data. Furthermore, the Asp metabolism pathway in the infected lungs were analyzed based on our former transcriptomic data. The RT-PCR results showed that the Asp metabolism pathway was enriched after PmCQ2 infection, indicating that Asp may play a role during PmCQ2 infection (Supplementary Figure 5). Together, these data indicated that AA inhibited PmCQ2 virulence factor expression and bovine PmA infection causes AA deficiency.

### Supplementation With AA Reduces Bacterial Loads and Increases Survival Rates of Mice

**Figure 4:**
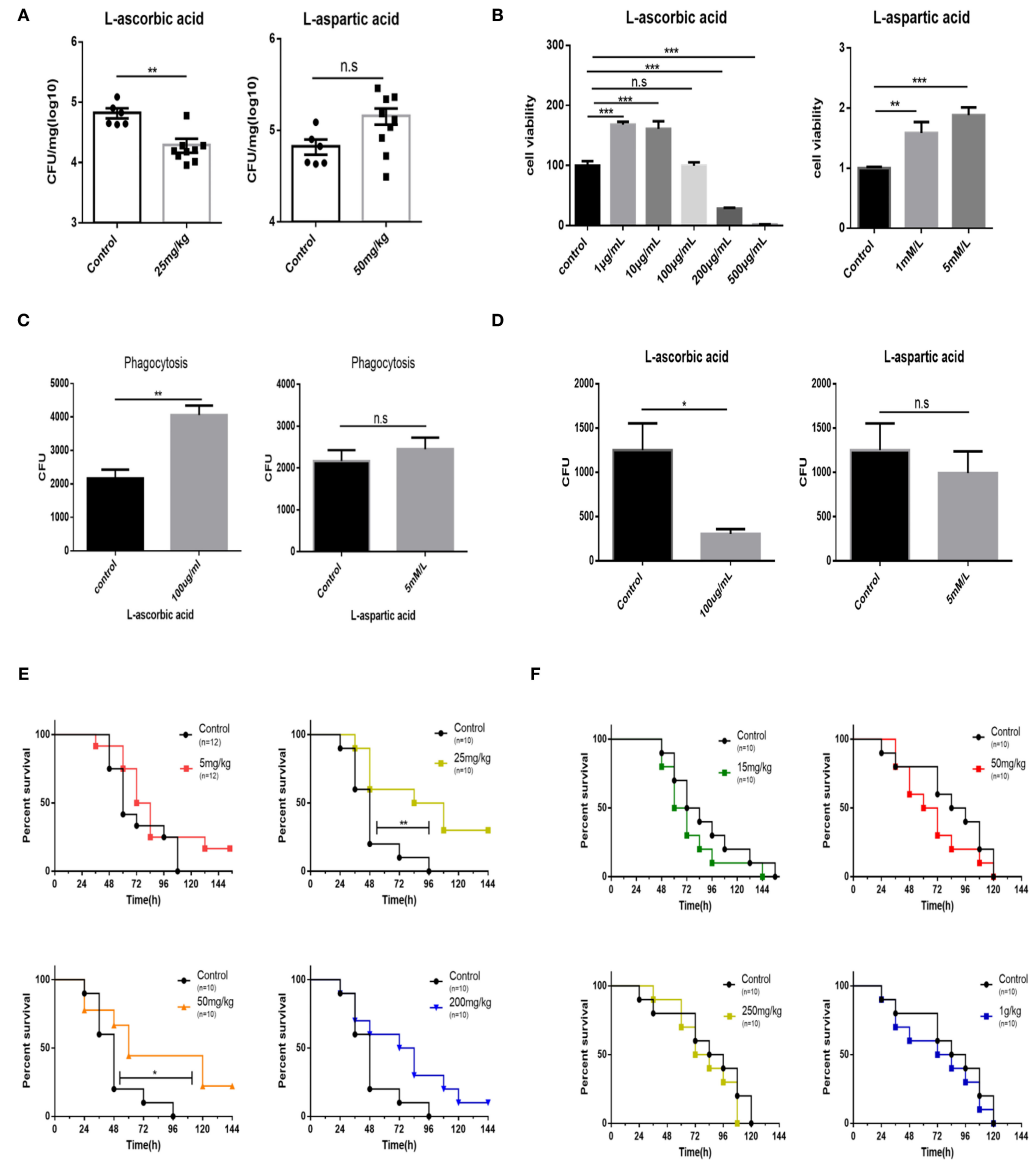
Supplementation of AA results in reduction bacteria density of the lung, promotion of macrophage bactericidal activity, and partial prevention of mice from death. **(A)** The bacteria density of the mouse lung infected with 10^4^ CFU log-phase growth PmCQ2 for 16 h. Mice were supplemented with AA, Asp, or saline for 7 days before infection. Every point indicates an individual. **(B)** Analysis of mouse peritoneal macrophage cell viability under different concentrations of AA or Asp. Five repeats for each group. **(C)** The phagocytosis of mouse peritoneal macrophage on PmCQ2. Cells were supplemented with 100 μg/ml AA, 5 mM Asp, or saline. Five repeats for each group. **(D)** The number of live PmCQ2 in mouse peritoneal macrophage. Five repeats for each group. **(E)** The survival rates of mice supplemented with different doses of AA or saline for 7 days. Mice were intraperitoneally infected with 10^4^ CFU log-phase growth PmCQ2. *n* represents how many mice were used in one experiment. **(F)** The survival rates of mice supplemented with different doses of Asp or saline for 7 days. Mice were intraperitoneally infected with 10^4^ CFU log-phase growth PmCQ2. *n* represents how many mice were used in one experiment. *, **, and *** represent *P* < 0.05, *P* < 0.01, and *P* < 0.001, respectively.

Afterwards, it is speculated that supplementation with exogenous AA can shape bovine PmA infection. Unsurprisingly, supplementation with AA reduces the bacterial burden of the infected lungs, demonstrating that exogenous AA acts as an anti-infection function (Figure 4A). On the contrary, supplementation of Asp is unable to change the bacterial density of the infected lung (Figure 4A). Moreover, although supplementation with Asp had no impact on mice mortality, supplementation with AA partially prevented infected mice from death, demonstrating that AA had a protective role during bovine PmA infection (Figures 4E,F). To further investigate the potential mechanism by which exogenous AA reduces bacterial density, we speculated that macrophage, which is responsible in clearing invaders *via* phagocytosis, might be involved in this process. First, various concentrations of AA and Asp were added to test the influence on the viability of macrophage. It is identified that the maximum concentration of AA without influencing macrophage viability is 100 μg/ml, which is consistent with a published paper (30), while Asp showed non-cytotoxicity in this work (Figure 4B). Next, as shown in Figures 4C,D, AA enhanced macrophage phagocytosis and decreased intracellular bacterial survival, while Asp is ineffective. Thus, the above data indicated that AA executed an anti-infection function in bovine PmA infection.

## Discussion

*Pasteurella multocida* is a gram-negative pathogen and has various host types, including cattle, birds, pigs, cats, and dogs (31). Among them, bovine PmA is one of the most prevalent pathogen in cattle around the world, causing representative respiratory tract syndrome with high lethality (32, 33). In China, since 2008, the respiratory pathogen bovine PmA has replaced *P. multocida* serogroup B to become the most prevalent pathogen in clinics. Thus, preventing bovine PmA infection is of importance to most researchers. However, it is difficult for researchers to use cattle to investigate bovine *P. multocida* infection, because there are few available SFP cattle and the cost is also high for most researchers. To mimic *P. multocida* infection, most researchers selected mouse, and our previous work has demonstrated that the mouse model successfully mimics bovine *P. multocida* infection, showing similiar lung pathological changes with clinical samples (27, 34). In the present work, mice infected with PmCQ2, a bovine PmA strain, showed typical pneumonia symptoms, such as cough and abdominal respiration, and high lethality. Also, a serious inflammation reaction was further identified by H&E staining of the infected lungs (Figures 1A–C). Next, it is found that the lung had higher bovine PmA density than other tissues during infection, even though the bacterial density of the infected spleen had no significant difference with the infected lung in intraperitoneal and intramuscular injection groups (Figure 1D). In line with Collins' report, a bacteremia phenomenon was further identified in infected mice and cattle (our unpublished data), namely, spleen allows bacteria in the blood detained in the spleen *via* its blood filter function instead of colonization (Supplementary Figure 1) (11, 28). Thus, the lung was considered as the organ containing the highest bacterial density in this work.

An emerging problem has emerged: why does the lung have the highest bacterial density than others under bovine PmA infection? Generally, bacterial colonization is shaped by many factors, like virulence factor, host immunity, and nutrition. For example, Petrova and his co-workers reported that the virulence gene *LGR1_llp1* of *Lactobacillus rhamnosus* influences its colonization in the vagina and the mutant strain shows half colonization number compared with the wild type (35). In addition, L-glutamine affects the expression of *L. monocytogenes* virulence genes and its colonization number in tissues (15). In our previous works, we have found that some amino acids, such as L-serine, L-arginine, L-proline, and L-glutamine, are closely related to bovine PmA infection (17–20). To find out potential nutrition shaping bovine PmA infection, we compared the metabolite profiles of high- and low-bacterial density tissues of the infected lung and liver using metabolomics. A total of 99 differently expressed metabolites were found, of which AA and Asp showed direct impact on bovine PmA growth *in vitro* (Figures 2A,B and Supplementary Figure 4). More specifically, AA inhibited bovine PmA growth, which expressed more in the infected liver than the lung, while the L-aspartic acid, which expressed more in the infected lung than the liver, showed promotion on bovine PmA growth (Figure 2C). Various papers have reported that AA, also called vitamin C, has an antimicrobial activity. For instance, AA has been reported to inhibit *Staphylococcus aureus, Pseudomonas aeruginosa*, and *Enterococcus faecalis* growth (36). Mechanically, Vilchèze and his co-workers found that the AA operates its antimicrobial activity through disrupting bacterial Fenton reaction (37). In this study, it is found that the inhibitory effect of AA was conservative in bovine PmA as AA inhibits another bovine PmA PmCQ6 growth. Contrary to AA, few papers have reported that Asp promotes bacterial growth, and an example is that Asp enhances *Acetobacter pasteurianus* growth (38). Notably, a recent paper pointed out that the aspartate ammonia-lyase (aspA) in Pm is essential for bacterial growth *in vitro* (39).

Bacterial virulence gene expression is vital for bacterial colonization. In the present paper, it is found that AA regulates bovine PmA virulence factor expression. In order to verify whether AA and Asp directly regulated bovine PmA virulence gene expression, several virulence gene expressions were tested, including two identified in our lab, *pm0442* (40) and *pm0979* (unpublished data). As shown in Figures 3A,B, Asp has an irregular influence on the virulence gene expression, while AA repressed *OmpA* and *oma87* expressions, and especially for *OmpA*, its inhibitory effect was in a dose-dependent manner (Figures 3A,B). Noteworthy, *OmpA* serves as a very important structural gene and virulence gene for *P. multocida* and is closely associated with outer membrane stability, bacterial adhesion, and colonization (41, 42). In addition, bacterial biofilm formation is a bacterial virulence factor through which bacteria escape from monitoring of the immune system and drug treatment (29), and a previous research indicated that *OmpA* was related to bacterial biofilm formation in *P. multocida* (26). Thus, it is speculated that the PmA biofilm formation can be reduced by AA. As shown in Figure 3D, bovine PmA biofilm formation is inhibited by AA, which is also consistent with published papers showing AA has a negative regulation on bacterial biofilm formation (43, 44). Consistent with previous results that infected lung owns less AA than the infected liver, higher *OmpA* expression was detected in the infected lung than the infected liver (Figure 3C). Unlike humans who rely on exogenous AA to meet physiological needs, mouse and cattle are capable to synthetize enough AA for themselves through a special gene *Gulo*, which only expresses in the liver (45, 46). Downregulation of *Gulo* expression is then found in the infected liver, indicating that AA biogenesis was damaged under bovine PmA infection (Figure 3E). Also, AA deficiency caused by the downregulation of *Gulo* was further evident by the reduction of the lung AA concentration after bovine PmA infection. One possible reason is that the supply of AA for peripheral organs, like the lung, was impaired, resulting in the compromised AA biogenesis. Additionally, the metabolomics data showing that AA expressed more in the infected liver than in the infected lung were examined by our AA kit (Figure 3F). So far, no published data has reported whether bovine PmA infection can influence AA concentrations in cattle, but an early paper indicated that the concentrations of AA were decreased in chiken cholera, which is caused by *P. multocida* infection (47).

The present work further speculated that supplementation with AA or Asp may have an impact on PmCQ2 infection. As shown in Figure 4A, supplementation with AA significantly reduced the lung bacterial density while supplementation with Asp only has an upward trend on lung bacterial density. Additionally, various papers have demonstrated that AA has an impact on macrophage function, and macrophages played a vital role during *P. multocida* infection (48). For instance, intracellular accumulation of pharmacologic AA concentrations in human monocytes inhibited its apoptosis pathway activation (49). In addition, other studies also found that supplementation with AA enhances phagocytosis and low intracellular AA restricts the ability of macrophage to clean the extracellular bacteria (50–52). Mechanically, phagocytosis mainly operated by macrophage and neutrophil produces much reactive oxygen species (ROS) to kill microbes, and excessive ROS can be neutralized by intracellular AA (53, 54). Accordingly, excessive accumulation of ROS reduces intracellular AA concentrations and impedes phagocytosis (55). Although Asp had no significant effect on changing bacterial density, it was set as a positive control for AA. First, it is found that 100 μg/ml AA was the maximum concentration for macrophage without interfering its viability, which is in line with previous reprorts (30, 56), while Asp seems to have no toxicity for macrophagy at 5 mM. Subsequently, phagocytosis and intracellular survival assays showed that phagocytosis and clearance of PmCQ2 were enhanced by AA, while Asp was ineffective (Figures 4C,D), implying that AA reduces the bacterial density *via* enhancing macrophage phagocytosis. However, further work needs to identify whether there are other mechanisms. Lastly, as shown in Figures 4E,F, supplementation with Asp had no effect on mice survival rate at least in our trials, but supplementation with AA significantly improved mice survival rate. However, the impact of AA supplementation on survival rate was in a dose-independent manner, and higher AA supplementation seems to have less benefit for survival rate. One possible cause is that AA damages cell viability and host coagulation function at an undesirably high concentration (57). A series of papers have demonstrated that supplementation with AA has an anti-infection effect. For example, AA supplementation effectively enhances animal resistance to endotoxin and tetanus toxin (58, 59). Furthermore, researches reported that supplementation of AA is beneficial for many bacterial diseases, like *Helicobacter pylori* infection, *P. aeruginosa* infection, *Streptococcus pneumoniae* infection, bacterial peritonitis, and bacterial sepsis (60–64). In the field of veterinary medicine, for instance, Hamdy's group found that AA supplementation can improve the survival rate of sheep in spontaneous pneumonia, and Naresh's group demonstrated that supplementation of AA can improve the recovery rate of dairy cows with mastitis in clinic (65, 66). Although mice and cattle synthesize enough AA under physiological conditions, AA deficiency resulting from reduction of *Gulo* expression was observed under bovine PmA infection in the present paper. In addition, in clinic, bovine PmA cases are constantly seen in calves or cattle that have been subjected to long-time or long-distance transportation. Notably, calves, unlike cattle, are unable to sythetize enough AA by themselves (67), showing AA deficiency. Likewise, Padilla's paper indicated that cattle suffers an AA deficiency under heat stress, and studies on other mammals, such as horse and goat, also demonstrated that transportation stress causes AA deficiency (68–70). Hence, it is assumed that cattle suffering from transportation stress might also show AA denficiency, which, in turn, make cattle sensitive to bovine PmA infection.

Although our mouse model successfully mimics bovine PmA infection, and based on our mouse model, the present work found that L-ascorbic acid shaped bovine PmA infection. The lack of direct evidence from cattle is a considerable limitation of this work. In addition, our primary work failed to find one optimal dose of L-ascorbic acid for supplementation and did not test or compare the effect of other supplementation methods, such as drinking and feeding. Thus, considering the clinical status and the limitations of the present work, our lab intends to start a systematic clinic trial to test the effect of AA supplementation on bovine PmA infection in our further works and, at the same time, to verify the mechanism on bovine cell lines mentioned in this work.

In conclusion, the current study found that AA shapes bovine PmA infection through comparing high- and low-bacterial density tissues. Mechanically, AA inhibited bovine PmA growth as well as virulence expression and regulated macrophage bactericidal activity. Moreover, bovine PmA infection caused AA deficiency and supplementation with AA reduced the bacterial density of the lung and improved mice survival rates, making AA a potential agent for the prevention of this disease in clinic.

## Data Availability Statement

The original contributions presented in the study are included in the article/Supplementary Material, further inquiries can be directed to the corresponding author/s.

## Ethics Statement

The animal study was reviewed and approved by Ethics committee of Southwest University.

## Author Contributions

YP, NL, and GZ have designed these experiments. GZ participated in all experiments and wrote this manuscript. PL and HM helped GZ do these experiments and analyze data. PL, NL, HM, and GZ revised this article. YP and NL approved the final manuscript. All authors contributed to the article and approved the submitted version.

## Conflict of Interest

The authors declare that the research was conducted in the absence of any commercial or financial relationships that could be construed as a potential conflict of interest.
