# Corrigendum: L-ascorbic acid shapes bovine *Pasteurella multocida* serogroup A infection

**DOI:** 10.3389/fvets.2023.1281834

**Published:** 2023-09-12

**Authors:** Guangfu Zhao, Pan Li, Hao Mu, Nengzhang Li, Yuanyi Peng

**Affiliations:** ^1^Chongqing Key Laboratory of Forage and Herbivorce, College of Veterinary Medicine, Southwest University, Chongqing, China; ^2^Key Laboratory for Bio-Resource and Eco-Environment of Education of Ministry, The Center for Growth, Metabolism and Aging, College of Life Sciences, Sichuan University, Chengdu, China; ^3^Chongqing Academy of Animal Science, Chongqing, China

**Keywords:** Bovine *Pasteurella multocida* serogroup A, pneumonia, metabolomics, macrophage, L-ascorbic acid

In the published article, there was an error in the legend for **Figures 3A–D** as published. The figure part labels were mixed up and the wrong description was given of each letter (A–D). The legend previously stated:

“**(A)** Analysis of some PmCQ2 virulence gene expression under different doses of AA. **(B)** Analysis of some PmCQ2 virulence gene expression under different doses of L-aspartic acid. Three repeats for each group. **(C)** PmCQ2 bioflim formation treated with different doses of AA or L-aspartic acid. Five repeats for each group. **(D)** The virulence gene, *OmpA*, expressions in the mice lung before infection or after 10^4^ CFU log-phase growth PmCQ2 infection for 16 h. Three repeats for each group.”

The corrected legend appears below.

**“(A)** Analysis of some PmCQ2 virulence gene expression under different doses of L-aspartic acid. **(B)** Analysis of some PmCQ2 virulence gene expression under different doses of AA. Three repeats for each group. **(C)** The virulence gene, *OmpA*, expressions in the mice lung before infection or after 10^4^ CFU log-phase growth PmCQ2 infection for 16 h. Three repeats for each group. **(D)** PmCQ2 biofilm formation treated with different doses of AA or L-aspartic acid. Five repeats for each group.”

In the published article, there was an error. The part labels for **Figure 3** were mixed up and the wrong description was given of each letter (A-D).

A correction has been made to **Results**, *AA Inhibits PmCQ2 Virulence Factor Expression and Bovine PmA Infection Leads to AA Deficiency*, Paragraph 4. This sentence previously stated:

“As shown in **Figure 3B**, Asp showed no impact on the virulence gene expression, whereas AA downregulated two virulence genes, *OmpA* and *oma87*, in a dose-dependent manner (**Figure 3A**). Interestingly, in agreement with the metabolomics data, less AA was found in the infected lung than the liver with more *OmpA* expression (**Figure 3D**). In addition, it is found that AA significantly decreased PmCQ2 biofilm biogenesis while Asp promoted PmCQ2 biofilm biogenesis at low dosage (**Figure 3C**).”

The corrected sentence appears below:

“As shown in **Figures 3A, B**, Asp showed no impact on the virulence gene expression, whereas AA downregulated two virulence genes, *OmpA* and *oma87*, in a dose-dependent manner. Interestingly, in agreement with the metabolomics data, less AA was found in the infected lung than the liver with more *OmpA* expression (**Figure 3C**). In addition, it is found that AA significantly decreased PmCQ2 biofilm biogenesis while Asp promoted PmCQ2 biofilm biogenesis at low dosage (**Figure 3D**).”

A correction has been made to **Discussion**, Paragraph 3. This sentence previously stated:

“As shown in **Figure 3C**, bovine PmA biofilm formation is inhibited by AA, which is also consistent with published papers showing AA has a negative regulation on bacterial biofilm formation (**Figure 3C**) (43, 44). Consistent with previous results that infected lung owns less AA than the infected liver, higher *OmpA* expression was detected in the infected lung than the infected liver (**Figure 3D**).”

The corrected sentence appears below:

“As shown in **Figure 3D**, bovine PmA biofilm formation is inhibited by AA, which is also consistent with published papers showing AA has a negative regulation on bacterial biofilm formation (43, 44). Consistent with previous results that infected lung owns less AA than the infected liver, higher *OmpA* expression was detected in the infected lung than the infected liver (**Figure 3C**).”

The authors apologize for these errors and state that this does not change the scientific conclusions of the article in any way. The original article has been updated.

