# Supplementary material for: L-Ascorbic Acid Shapes Bovine Pasteurella multocida Serogroup A Infection
Source: Front Vet Sci. 2021 Jul 8;8:687922. doi: 10.3389/fvets.2021.687922 (PMC8295749; doi:10.3389/fvets.2021.687922)
Supplement: Supplementary file 1 [file Table_1.DOCX]

**List of primers used in this paper.**

Table S1 List of primers

| genes | 5’-3’ sequence | Size (bp) |
| --- | --- | --- |
| *OmpA* | Forward-AAACCATCAAGTAGCGGGTAG | 146bp |
|  | Reverse-AATTTCGCAGTAGAGTTAGGCTATG |  |
| *oma87* | Forward-CTTGCGGCATCCACAAACAA | 71bp |
|  | Reverse-GTCCCAACACCATTTGTCGC |  |
| *pm0979* | Forward-ACGAAACGCACACCATCTTGATAAG | 95bp |
|  | Reverse-GGTAAAGCAGATAGCGCAACAGTAACAAT |  |
| *pm0442* | Forward-TGGAAGAAGTGAAAGCCGCTACTG | 135bp |
|  | Reverse-CCATCTTTGTCGCCGTAGCAG |  |
| *TbpA* | Forward-CGAACAACGTTCTGCTCTCC | 187bp |
|  | Reverse-TCGCAGATAAACGATGGCGA |  |
| *PfhB2* | Forward-AACGCAGAGATTAGGAGTACGACAC | 138bp |
|  | Reverse-ACCATAAATACGCCCAGCATTAGT |  |
| *16S RNA* | Forward-ACGCTGGCGGCAGGCTTAAC | 101bp |
|  | Reverse-ATTCCCAAGCATTACTCACCCGTCC |  |
| *CAD* | Forward-AGGCATCACCCCAGAACCTA | 199bp |
|  | Reverse-GCTGCCAGCTTCAGGATGTC |  |
| *Asns* | Forward-CTCCAACCGGTCTTGTCACT | 74bp |
|  | Reverse-AGGCACTCTGAGCACTAGC |  |
| *Aspa* | Forward-TCTGCAGGATCAAGACTGGAA | 188bp |
|  | Reverse-GGATGCTTTTTGCGCTGAGT |  |
| *Folh1* | Forward-AAACTCTTAGAAAAGGTCAAGATGC | 91bp |
|  | Reverse-CCAGAGCTCCTTTGAGGGTG |  |
| *GOT1* | Forward-TCAGGGAGAATCGGGTTGGA | 195bp |
|  | Reverse-AGCAATAGGGCCGAATGTCC |  |
| *4930571K23Rik* | Forward-CATGGGAACCAAGCTGACCA | 117bp |
|  | Reverse-GGGGCAGTGGGATTGGTTTA |  |
| *Nat8l* | Forward-CGCAAGGTGATTCTGGCCTA | 97bp |
|  | Reverse-CCCAGAAACAGGAACCAGGTG |  |
| *Gulo* | Forward-TGGGCAAAGGCCCACAAT | 160bp |
|  | Reverse-GCAGCTTGCTTCTGCTTCTAC |  |
| *Beta-actin* | Forward-CACTGTCGAGTCGCGTCC | 89bp |
|  | Reverse-TCATCCATGGCGAACTGGTG |  |
