# Supplementary material for: L-Ascorbic Acid Shapes Bovine Pasteurella multocida Serogroup A Infection
Source: Front Vet Sci. 2021 Jul 8;8:687922. doi: 10.3389/fvets.2021.687922 (PMC8295749; doi:10.3389/fvets.2021.687922)
Supplement: Supplementary file 2 [file Table_2.DOCX]

**The list of differentially expressed metabolites**

Differentially expressed metabolites

| Name | Average Mean (Infected Liver) | Average Mean (Infected Lung) |
| --- | --- | --- |
| 1,5-Anhydroglucitol | 13.99038176 | 0.337441124 |
| nicotinamide | 6.855226447 | 4.198663407 |
| ascorbate | 5.314722311 | 3.514881924 |
| allose 1 | 5.280341148 | 0.263990582 |
| succinic acid | 3.601956629 | 9.98549E-06 |
| beta-Alanine 2 | 2.870276822 | 0.827125565 |
| Fructose 2,6-biphosphate degr prod 2 | 1.740488592 | 0.53078529 |
| glucuronic acid 2 | 1.114511762 | 9.98549E-06 |
| 4-aminobutyric acid 3 | 0.924169207 | 0.223930176 |
| 3-Methylamino-1,2-propanediol 1 | 0.56730055 | 0.008271067 |
| Carnitine | 0.518650816 | 9.98549E-06 |
| flavin adenine degrad product | 0.292935558 | 0.194928861 |
| Digitoxose 2 | 0.292240891 | 9.98549E-06 |
| Pyruvic acid | 0.274561707 | 9.98549E-06 |
| alanine 2 | 0.221303351 | 0.08682779 |
| adipic acid | 0.124141914 | 0.008152918 |
| gluconic acid 1 | 0.067126075 | 0.002086095 |
| D-erythro-sphingosine 1 | 0.041448472 | 9.98549E-06 |
| urocanic acid 2 | 0.034081041 | 9.98549E-06 |
| Isomaltose 2 | 0.033744218 | 9.98549E-06 |
| Carbobenzyloxy-L-leucine | 0.033463873 | 9.98549E-06 |
| 6-hydroxy caproic acid | 0.029182568 | 9.98549E-06 |
| N(epsilon)-Trimethyllysine N | 0.027370768 | 0.012422792 |
| L-Threose 1 | 0.02430559 | 9.98549E-06 |
| Sophorose 2 | 0.023137146 | 9.98549E-06 |
| Cysteinylglycine 2 | 0.020599281 | 9.98549E-06 |
| N-Methyl-L-glutamic acid 2 | 0.018958378 | 9.98549E-06 |
| threonine 1 | 0.00721601 | 9.98549E-06 |
| 3-hydroxy-L-proline 1 | 0.00453423 | 9.98549E-06 |
| oxalic acid | 25.22152249 | 81.88973484 |
| oxamic acid | 23.75617666 | 48.12933259 |
| aspartic acid 1 | 8.12580961 | 38.80818769 |
| Methyl Phosphate | 16.10301443 | 21.67040327 |
| proline | 14.42971405 | 19.50495877 |
| stearic acid | 13.09888557 | 19.38312038 |
| creatine | 0.827846887 | 18.89953212 |
| valine | 13.2661606 | 17.92410503 |
| hydroxyurea | 2.05003057 | 16.16897663 |
| Itaconic acid | 1.31186938 | 16.16553881 |
| Benzoin 2 | 8.040661796 | 10.69939889 |
| beta-Glutamic acid 1 | 0.532945247 | 9.59141484 |
| Aminomalonic acid | 1.859908505 | 9.530887337 |
| L-Allothreonine 1 | 2.555865941 | 7.045981357 |
| Phenylphosphoric acid | 3.77497585 | 6.345998918 |
| phosphomycin | 1.748750644 | 5.255176797 |
| fumaric acid | 2.69909719 | 4.73544247 |
| palmitic acid | 2.391576621 | 4.720436076 |
| glutamic acid | 1.507112786 | 4.637516672 |
| Lactamide 1 | 0.82872141 | 3.573750924 |
| Threonic acid | 0.477571613 | 3.471451048 |
| citric acid | 0.246644215 | 2.980992475 |
| 1,2-Cyclohexanedione 2 | 1.343538759 | 2.54536165 |
| N-epsilon-Acetyl-L-lysine 1 | 0.056774892 | 2.388294658 |
| Myristic Acid | 4.26679E-06 | 2.166421806 |
| malonic acid 1 | 0.434880042 | 1.476268486 |
| methionine 1 | 0.695482669 | 1.13235441 |
| 2-ketoadipate 3 | 0.221297722 | 1.111194543 |
| uracil | 0.169484614 | 1.069671292 |
| lactic acid | 4.26679E-06 | 0.921500432 |
| N-acetyl-L-aspartic acid 1 | 0.019848794 | 0.756981342 |
| 3-Aminoisobutyric acid 1 | 0.271789754 | 0.637354951 |
| D-Glyceric acid | 0.159622016 | 0.611207568 |
| Methyl Palmitoleate | 4.26679E-06 | 0.582897694 |
| 2,3-Dihydroxypyridine | 0.301392034 | 0.526175822 |
| cis-Phytol | 0.003058706 | 0.520496545 |
| 21-hydroxypregnenolone 2 | 0.118696646 | 0.472597412 |
| 2-Deoxyuridine | 4.26679E-06 | 0.388669561 |
| 3-hydroxybutyric acid | 0.198521903 | 0.377409788 |
| Androstanediol | 0.27140482 | 0.361683188 |
| 4-Acetamidobutyric acid 2 | 0.05123798 | 0.305273688 |
| O-acetylserine 1 | 4.26679E-06 | 0.297540753 |
| 2-keto-isovaleric acid 1 | 0.073712206 | 0.205183076 |
| pantothenic acid | 0.042971381 | 0.184952407 |
| palmitoleic acid | 0.050505496 | 0.184907291 |
| D-Arabitol | 0.029225355 | 0.152789822 |
| isocitric acid 2 | 4.26679E-06 | 0.150813601 |
| thymine | 0.001607563 | 0.148970822 |
| glutamine 3 | 0.041027914 | 0.147817269 |
| 3-Hydroxypropionic acid 1 | 0.058426625 | 0.126806028 |
| 2'-Deoxycytidine 5'-triphosphate degr prod | 4.26679E-06 | 0.099532639 |
| terephthalic acid | 0.006487082 | 0.095301206 |
| catechol | 0.007498978 | 0.095011081 |
| tartaric acid | 0.048768293 | 0.089880179 |
| cis-gondoic acid | 0.002807355 | 0.087345742 |
| L-homoserine 1 | 0.037449489 | 0.082757136 |
| fructose 1 | 0.003045595 | 0.074357339 |
| Glutaric Acid | 0.00218686 | 0.074110378 |
| N-Acetyl-D-galactosamine 1 | 4.26679E-06 | 0.073924408 |
| 3-Phenyllactic acid | 0.007221523 | 0.073502417 |
| Methyl jasmonate 3 | 0.012975948 | 0.073134628 |
| cuminic alcohol | 4.26679E-06 | 0.070529469 |
| hydroxylamine | 4.26679E-06 | 0.041020966 |
| sorbitol | 4.26679E-06 | 0.036680039 |
| 3-(2-Hydroxyphenyl)propionic acid | 4.26679E-06 | 0.032662634 |
| 2-Methylglutaric Acid | 4.26679E-06 | 0.02239595 |
| 3,5-Dihydroxyphenylglycine 1 | 4.26679E-06 | 0.019611293 |
| farnesol 1 | 4.26679E-06 | 0.014339957 |
| 6-Methylmercaptopurine 2 | 4.26679E-06 | 0.011430738 |
| maleic acid | 0.000340588 | 0.007167456 |
