# Supplementary material for: L-Ascorbic Acid Shapes Bovine Pasteurella multocida Serogroup A Infection
Source: Front Vet Sci. 2021 Jul 8;8:687922. doi: 10.3389/fvets.2021.687922 (PMC8295749; doi:10.3389/fvets.2021.687922)
Supplement: Supplementary file 3 [file Data_Sheet_1.DOCX]

**Figure S1. Identification of *Pasteurella multocida* serogroup A in the infected mouse tissues and heart blood based on PCR results.** KMT1 positive means this bacterium belongs to *pasteurella* *multocida* species and *hyaD-hyaC* positive means this bacterium belongs to serogroup A.


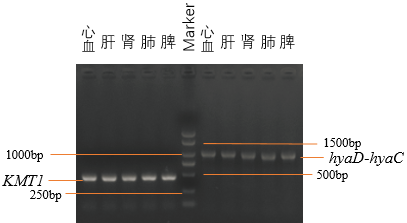


Heart blood

The lung

The liver

The spleen

The kidney

Marker

Heart blood

The lung

The liver

The spleen

The kidney
