# Supplementary material for: L-Ascorbic Acid Shapes Bovine Pasteurella multocida Serogroup A Infection
Source: Front Vet Sci. 2021 Jul 8;8:687922. doi: 10.3389/fvets.2021.687922 (PMC8295749; doi:10.3389/fvets.2021.687922)
Supplement: Supplementary file 4 [file Data_Sheet_2.docx]

**Supplementary Figure S2.** A. The bacteria load of the mouse lung after infected with 10^4^ CFU log-phase growth DH5α for 32h. B. The bacteria density of the mouse lung and liver infection with 10^4^ CFU log-phase growth bovine mastitis *E. coli* and *Salmonella typhimurium* for 48h.


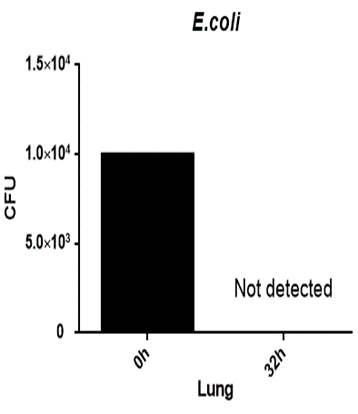


**A**


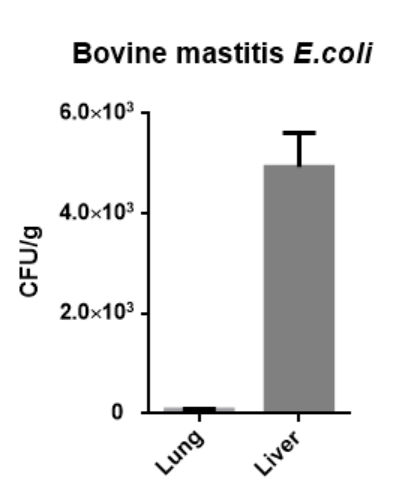

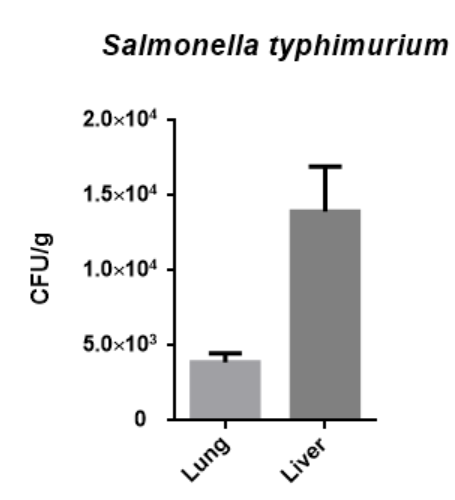


**B**
