# Supplementary material for: L-Ascorbic Acid Shapes Bovine Pasteurella multocida Serogroup A Infection
Source: Front Vet Sci. 2021 Jul 8;8:687922. doi: 10.3389/fvets.2021.687922 (PMC8295749; doi:10.3389/fvets.2021.687922)
Supplement: Supplementary file 5 [file Data_Sheet_3.DOCX]

**Supplementary Figure S3.** Examination of metabolomics data. A. The principal components analysis (PCA). PCA is generated by the data of infected livers and infected lungs (SIMCA14.1 software package) and the majority of the samples are within the 95% confidence interval (Hotelling's T-squared ellipse). B. The orthogonal partial least-squares discriminant analysis (OPLS-DA). The parameters for the classification from the software were R^2^Y =0.998 and Q^2^Y = 0.973, which were stable and good to fitness and prediction. C. Permutation test of OPLS-DA model. The R2 and Q^2^ intercept values were (0,0.78) and (0,-0.93) after 200 permutations. The low values of Q^2^ intercept indicate the robustness of the models, and thus show a low risk of over fitting and reliable. Class 1 and Class 2means the mice infected livers and lungs, respectively.


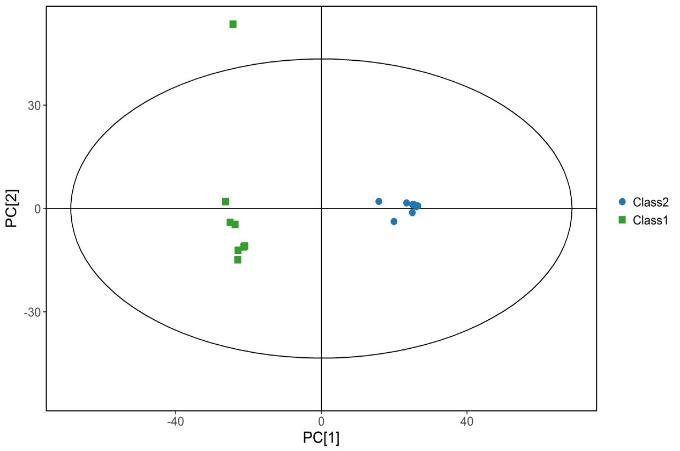


**A**


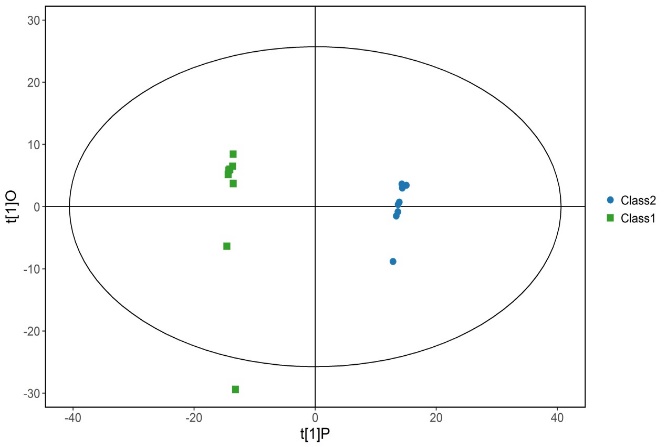


**B**


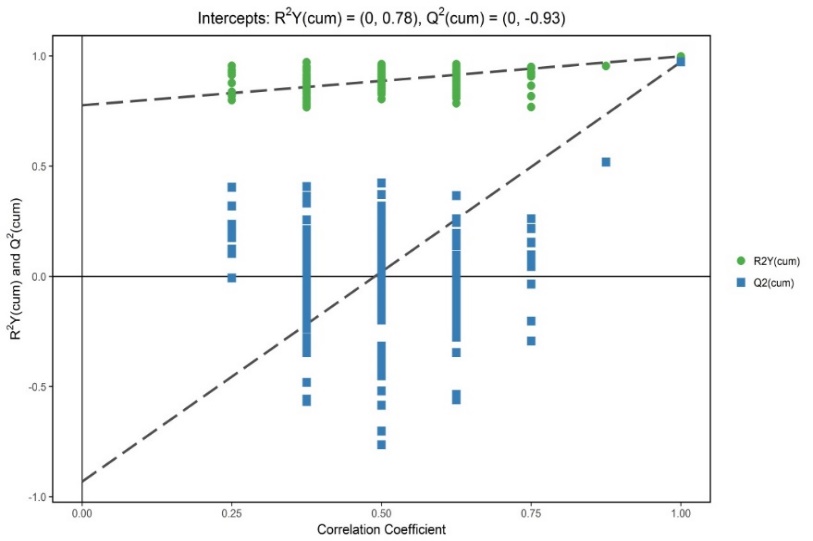


**C**
