# Supplementary material for: L-Ascorbic Acid Shapes Bovine Pasteurella multocida Serogroup A Infection
Source: Front Vet Sci. 2021 Jul 8;8:687922. doi: 10.3389/fvets.2021.687922 (PMC8295749; doi:10.3389/fvets.2021.687922)
Supplement: Supplementary file 6 [file Data_Sheet_4.DOCX]

**Supplementary Figure S4.** PmCQ2 impedance curves under different metabolites supplementation, which were measured by BacTrac™ 4300 Microbiological Analyzer. These metabolites showed no effect on PmCQ2 growth.

**
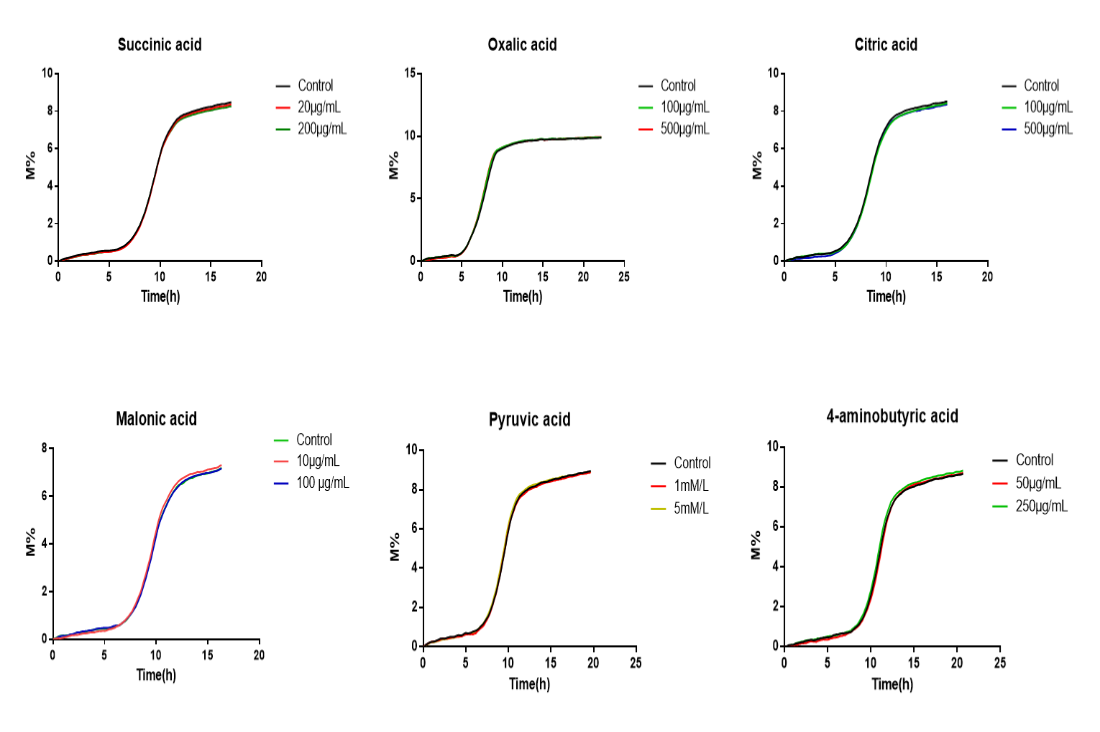
**
