# Supplementary material for: L-Ascorbic Acid Shapes Bovine Pasteurella multocida Serogroup A Infection
Source: Front Vet Sci. 2021 Jul 8;8:687922. doi: 10.3389/fvets.2021.687922 (PMC8295749; doi:10.3389/fvets.2021.687922)
Supplement: Supplementary file 7 [file Data_Sheet_5.DOCX]

**Supplementary Figure S5.** Asp metabolism pathway were riched in the infected lung of mice. A. Q-PCR results of Aspassociated metabolic enzymes. B. The picture of Asp metabolism pathway. The red, green and white reprents that the gene was up-regulated, down-regulated and no change in the infected lung, respectively.


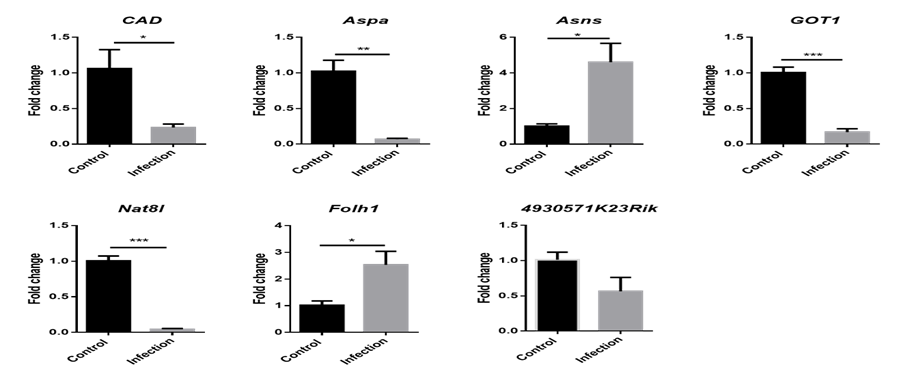


**A**


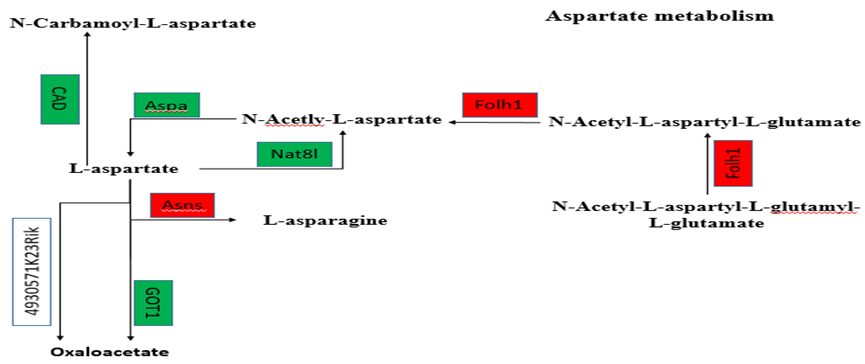


**B**
