# Supplementary material for: L-Ascorbic Acid Shapes Bovine Pasteurella multocida Serogroup A Infection
Source: Front Vet Sci. 2021 Jul 8;8:687922. doi: 10.3389/fvets.2021.687922 (PMC8295749; doi:10.3389/fvets.2021.687922)
Supplement: Supplementary file 8 [file Data_Sheet_6.DOCX]

**Materials and methods**

**Metabolomics protocol**

**Metabolites Extraction**

Tissues were collected aseptically and taken into sterile 4ml EP tubes. In order to avoid the randomness of data, each group was consisted with individual 8 tissues. Tissues were homogenized (35Hz for 4min) and ultrasoninc treatment (5min) incubated in ice water. Then, we collected the supernatant 0.21mL after centrifuging (3000rpm for 5min, 4℃) into 2ml tubes with 20μL of L-2-Chlorophenylalanine (1mg/mL) and mixed them. One more centrifuge (13000rpm for 15min, 4℃) was done and we uesd a fresh 2mL GC/MS glass vial to collected the supernatant (0.2mL), and dried them without heating. After that, 60μL Methoxy amination hydrochloride (20mg/mL in pyridine) and 80μL of the BSTFA regent (1% TMCS, v/v) was added in turn and were incubated for 30min (80℃) and 1.5h (70℃).

**GC-TOF-MS Analysis**

Samples were analysed by GC-TOF-MS system. This system is consisted of an Agilent 7890 gas chromatograph system and a Pegasus 4D time-of-flight mass spectrometer.

**Data preprocessing and annotation**

Utilizing LECO-Fiehn Rtx5 database and Chroma TOF 4.3X software (LECO Corporation) analyze these data, such as calibration of the baseline (Kind et al., 2009). Considerating both of mass spectrum match and retention index match ensure the metabolites identification.

**Metabolomics analysis**

First, using SIMCA14.1 software package (V14.1, MKS Data Analytics Solutions, Umea, Sweden) analyzed these data and then built the principal component analysis (PCA) and orthogonal projections to latent structures-discriminate analysis (OPLS-DA) to evaluate these samples. Second, utilizing 7-fold cross validation and permutation test to check whether our model was robustness and predictive. Based on P value and variable importance in the projection (VIP), the determinant of whether those data were selected as changed metabolites is P < 0.05 and VIP > 1.
